# Parasitic plants of the genus *Cuscuta* and their interaction with susceptible and resistant host plants

**DOI:** 10.3389/fpls.2015.00045

**Published:** 2015-02-04

**Authors:** Bettina Kaiser, Gerd Vogg, Ursula B. Fürst, Markus Albert

**Affiliations:** ^1^Institute of Plant Biochemistry, Centre for Plant Molecular Biology, University of TübingenTübingen, Germany; ^2^Department of Botany II – Ecophysiology and Vegetation Ecology, Julius-von-Sachs-Institut für Biowissenschaften, Botanischer Garten der Universität Würzburg, University of WürzburgWürzburg, Germany

**Keywords:** parasitic plants, plant–plant interaction, *Cuscuta*, dodder, plant immunity, resistance, symbiosis

## Abstract

By comparison with plant–microbe interaction, little is known about the interaction of parasitic plants with their hosts. Plants of the genus *Cuscuta* belong to the family of Cuscutaceae and comprise about 200 species, all of which live as stem holoparasites on other plants. *Cuscuta* spp. possess no roots nor fully expanded leaves and the vegetative portion appears to be a stem only. The parasite winds around plants and penetrates the host stems via haustoria, forming direct connections to the vascular bundles of their hosts to withdraw water, carbohydrates, and other solutes. Besides susceptible hosts, a few plants exist that exhibit an active resistance against infestation by *Cuscuta* spp. For example, cultivated tomato (*Solanum lycopersicum*) fends off *Cuscuta reflexa* by means of a hypersensitive-type response occurring in the early penetration phase. This report on the plant–plant dialog between *Cuscuta* spp. and its host plants focuses on the incompatible interaction of *C. reflexa* with tomato.

## INTRODUCTION

Plants live in a world populated by numerous and varied herbivores and microbial pathogens that include insects, nematodes fungi, bacteria, and oomycetes. However, plants have evolved mechanisms to detect such attacks and counteract them with efficient immune responses. Much of our knowledge about pathogen recognition of plants originates from studies of plant–microbe or plant–insect/herbivore interactions, in which plant immunoreceptors detect specific microbe-associated molecular patterns (MAMPs) that are often highly conserved microbial structures, such as bacterial flagellin or fungal chitin. Activation of these immunoreceptors, also termed pattern recognition receptors (PRR), triggers a set of typical defense responses that include rapid production of reactive oxygen species (ROS-burst), elevation of the stress-related phytohormone ethylene, increased levels of secondary metabolites (callose, phytoalexins, lignins, etc.) and the induction of characteristic marker genes (Boller and Felix, 2009; Böhm et al., 2014). Additionally, plant defense responses include signaling via networks controlled by the phytohormones salicylic acid (SA) and jasmonic acid (JA; Dong, 1998; Wasternack et al., 2006). In particular, SA is required for the initiation of a hypersensitive response (HR) and to trigger systemic acquired resistance (SAR; Durrant and Dong, 2004). Taken together, plants present a complex network of defense reactions to fend off pathogens or at least to restrict the pathogen growth and spread.

Apart from microbial pathogens and herbivorous arthropods, plants are also parasitized by other plants. Parasitic plants feed on their hosts (from the Greek *para* = beside; *sitos* = food) and keep them alive until they have completed their life cycle. Most often the parasite’s life cycle is completed earlier than the one of the host plant which leads to a premature death of the parasitized plants and thus can cause crop damage. Parasitic plants are categorized as either obligate or facultative parasites, depending on whether they rely totally on their hosts to complete their life cycle (obligate) or are able to survive on their own in the absence of their host plants (facultative). Additionally, parasitic plants can be divided into hemiparasites that rely only partially on a host plant and are still able to make photosynthesis and holoparasites that are completely dependent on photoassimilates, solutes, and metabolites from their host plants. According to their preferred target host organ, parasitic plants are defined as either root or shoot parasites.

Here, we describe the parasitic plant *Cuscuta* spp.—also known as dodder—which can be defined as an obligate stem holoparasite. Besides describing its life style and mechanisms for infecting susceptible host plants, we will focus on *Cuscuta* spp. as pathogens. Using mainly tomato vs. *Cuscuta reflexa* as an example, resistance mechanisms of plants against plant parasites will be illustrated and discussed, highlighting future prospects of controlling *Cuscuta* infestations during crop cultivation.

## *Cuscuta* ssp. – OCCURRENCE AND AGRONOMICAL ROLE

**FIGURE 1:**
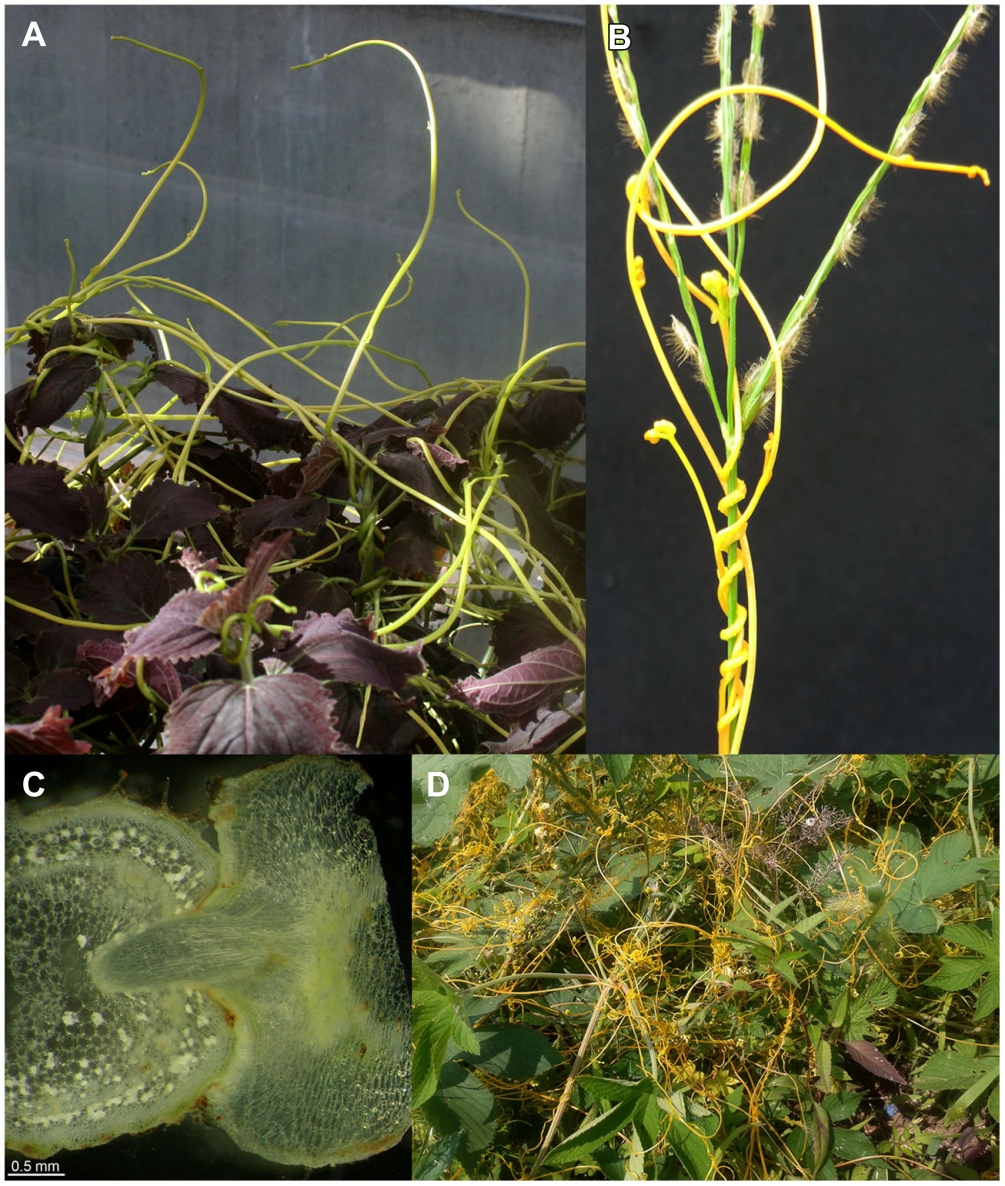
***Cuscuta* spp. on susceptible host plants. (A)** *Cuscuta reflexa* on *Coleus blumei*; cultivation in the greenhouse, University of Tübingen. **(B,D)** *Cuscuta australis* near Daejin-University, Pocheon, South Korea. **(B)** *C. australis* infecting a grass (monocotyledon). **(C)** Cross section of a *C. reflexa* haustorium growing into the shoot of a susceptible host plant (*Nicotiana benthamiana*) **(D)** *C. australis* on a dicotyledonous host plant.

Among the flowering plants, there are approximately 3,900 known parasitic plant species in more than 20 plant families (Westwood et al., 2010). Well-known and agriculturally important genera include *Striga* and *Orobanche* from the *Orobanchaceae* family and *Cuscuta* spp. (**Figure 1**) from the family of *Convolvulaceae*. While *Striga* and *Orobanche* can severely affect crop yields in drier and warmer areas of Africa and Asia (Spallek et al., 2013), *Cuscuta* spp. thrive in regions with a warm and more humid climate where the highest *Cuscuta*-dependent crop yield losses also occur (Dawson et al., 1994). Nevertheless, *Cuscuta* species can be found on all continents; for example, five species are native to central Europe (Mabberley, 1997), of which *C. europaea* is the most prominent. Agriculturally, the most important *Cuscuta* species are *C. pentagona* and *C. campestris*, which show an almost worldwide distribution and have a wide host spectrum. Severe crop loss due to *Cuscuta* is reported for 25 crop species in 55 countries (Lanini and Kogan, 2005). Highest species diversity for dodder occurs in the Americas, from Canada to Chile (Yuncker, 1932; Stefanovic et al., 2007). In Chilean crops, *C. chilensis*, *C. racemosa* var. *chiliana*, and *C. pentagona* are important. The biogeography of *Cuscuta* has recently been studied using plastid protein-coding (*rbcL)* and nuclear large-subunit ribosomal DNA (*nrLSU)* sequences covering the morphological, physiological, and geographical diversity of the genus (Garcia et al., 2014). This work supports the classical subgenera *Monogynella* and *Grammica* as monophyletic, leaving a paraphyletic subgenus *Cuscuta*, with the section *Pachystigma* as sister to the section *Grammica.* However, as *Cuscuta* is the only parasitic genus in the *Convolvulaceae* family, there is high similarity among the species within this genus (Garcia et al., 2014).

## *Cuscuta* LIFE CYCLE

Parasitic plants of the genus *Cuscuta* have no chlorophyll, or only a reduced amount, and are not usually photosynthetically active (Kuijt, 1969; Hibberd et al., 1998; Garcia et al., 2014). Only a few *Cuscuta* species still show residual photosynthesis (Dawson et al., 1994; Hibberd et al., 1998) and have thus been designated as cryptically photosynthetic (Funk et al., 2007; McNeal et al., 2007a,b). However, all *Cuscuta* species depend (absolutely) on a host plant to complete their life cycle, and *Cuscuta* can be considered an obligate holoparasite.

Like other angiosperms, the life cycle of *Cuscuta* begins with seed germination. Germinating *Cuscuta* seedlings depend on limited seed reserves; they are unable to survive alone for a longer time and must find an appropriate host plant stem within the first few days. To find and catch potential hosts, *Cuscuta* recognizes plant volatiles as chemo-attractants which guide seedling growth and increase the chances of successful infection (Runyon et al., 2006). In the presented study, the authors worked with *Cuscuta pentagona* seedlings and tomato plants as hosts. A detailed analysis identified the volatile terpenoids α-pinene, β-myrcene, and β-phellandrene as chemical cues that are produced by the tomato and serve as chemo-attractants for *Cuscuta*. As confirmed by our own observations of *C. reflexa*, the parasite does not distinguish between plant stems and sticks consisting of wood, metal, or plastic. The parasite winds around, totally unimpressed, and even develops (pre-) haustoria and tries to penetrate these victim-dummies. However, this was observed only with *C. reflexa* shoot parts taken from adult plants and not with *C. reflexa* seedlings. Runyon et al. (2010), in turn, used only *C. pentagona* seedlings, and could see no directed seedling growth to control stems such as artificial plants or vials of colored water (Mescher et al., 2006). In general, both detection of eventual light and chemical cues would require highly sensitive perceptual systems in *Cuscuta* ssp., but to date no such systems or receptors have been identified.

After finding an appropriate host plant, the first physical contact initiates an attachment phase, in which the parasitic epidermal and parenchymal cells begin to differentiate into a secondary meristem and develop prehaustoria, also known as adhesive disk (Dörr, 1968; Heidejorgensen, 1991). Important signals initiating and controlling this prehaustoria formation include mechanical pressure, osmotic potentials, and phytohormones such as cytokinins and auxin (Dawson et al., 1994; Runyon et al., 2010). The prehaustorial cells start to produce and secrete adhesive substances such as pectins and other polysaccharides, reinforcing the adhesion (Vaughn, 2002). During this attachment phase, host cells in proximity to the *Cuscuta* haustoria respond with an increase in cytosolic calcium, detectable in host plants expressing aequorin as calcium reporter. This increase lasts for about 48 h after the initial contact (Albert et al., 2010a,b). Cytosolic calcium signals are part of several signal transduction pathways, initiated by diverse stimuli such as touch, osmotic signals, phytohormones, or defense triggers. Since these signals could all be part of the plant–plant interaction, it is difficult as yet to assign a clear role for the described calcium spikes. Within the first hours of contact, *Cuscuta* also induces the host plant to produce its own sticky substances, such as arabinogalactan proteins, to promote adhesion (Albert et al., 2006). These glycoproteins are secreted by the host plant and localize to the cell-wall where they can force the adhesion together with other sticky components such as pectins. However, the initial signals from the parasite that trigger cellular responses in the hosts to mediate adhesion or consequent susceptibility are as yet unknown.

The attachment phase is followed by the penetration phase as prehaustoria develop into parasitic haustoria that penetrate the host stem through a fissure. This breach is effected by mechanical pressure (Dawson et al., 1994) and is supported by the biochemical degradation of host cell walls, caused by secreted hydrolytic enzymes such as methylesterases (Srivastava et al., 1994) or complexes of lytic enzymes consisting of pectinases and cellulases (so-called “loosening particles”; Vaughn, 2003). Cells at the tip of the invading haustoria (**Figure 1C**) form “searching hyphae,” which try to reach phloem or xylem cells of the host plant’s vascular bundles. After contact with a sieve cell, the searching hyphae grow around the cell like the fingers of a hand, and the parasitic cell surface interacting with the host sieve cell is enlarged more than 20 times. These parasitic cells have been described as having ambivalent characters, functioning as both sieve elements and transfer cells (Dörr, 1968, 1969, 1972; Dawson et al., 1994). Interestingly, during this process, chimeric cell walls of host and parasite constituents are formed, and interspecific plasmodesmata build up a cytoplasmic syncytium between *Cuscuta* and the host plant (Haupt et al., 2001; Vaughn, 2003; Birschwilks et al., 2006). To form a connection to the xylem, parasitic and host cells of the xylem parenchyma commence a synchronized development, fusing to build a continuous xylem tube from the host to the parasite (Dörr, 1972). With functional connections to the xylem and phloem of its host, the parasitic plant is supplied with water, nutrients, and carbohydrates (Jeschke et al., 1994, 1997; Hibberd et al., 1999; Hibberd and Jeschke, 2001).

The haustorium (from Latin “haurire” = to drink) is the defining feature of all parasitic plants, representing the interface where nutrients, solutes, and carbohydrates are exchanged between host and parasite (Chang and Lynn, 1986; Dawson et al., 1994; Hibberd et al., 1999; Yoshida and Shirasu, 2009; Westwood et al., 2010). This interface also allows the exchange of macromolecules between the host plant and the parasite. For example, it has been demonstrated that phloem-mobile GFP, a 25 kDa protein, can be successfully transferred from the host plant to *Cuscuta* (Haupt et al., 2001), probably by passing through the interspecific plasmodesmata. The transfer of viruses between parasite and host had already been described in 1947 (Sakimura, 1947); more recent publications report findings about the uptake of host RNAs by the parasite (Roney et al., 2007; David-Schwartz et al., 2008; Westwood et al., 2009). This phenomenon was also used in a biotechnological approach to cross-species transfer of small RNAs for the control of crop parasites (Runo et al., 2011)—specifically, of *Cuscuta* infestation (Alakonya et al., 2012)—and will be discussed in detail later. Very recently, it was shown that transcripts are transferred in a bidirectional manner between *C. pentagona* and susceptible host plants (Kim et al., 2014). In the presented study, the authors infected stems of tomato (*Solanum lycopersicum*) and *Arabidopsis thaliana* with *C. pentagona,* and isolated and sequenced RNA from the infection site, the host stem above the region of attachment and the parasite stem near the region of attachment. While it was expected that RNAs would move from the host into the parasite, the efficiency of this process was indeed surprising; almost half of the *A. thaliana* transcriptome was detectable in *C. pentagona* stem sections close to the haustoria. It was also astonishing that transcripts did not move only unidirectionally from host to parasite, but that parasitic transcripts were found in the host stems, just a few centimeters away from the infection site. The role of this bidirectional transcript exchange between plants is wholly unclear; one could speculate whether RNAs (especially small RNAs) might have a signaling function in hosts or parasites. It remains to be shown whether this interchange of RNAs has anything to do with horizontal gene transfer; a *Cuscuta* genome sequencing project would shed more light on such mechanisms. However, the impressive extent of exchanged macromolecules such as RNAs confirms the haustorium as an “open door” between host and parasite that seems not especially guarded.

## INTERACTION OF *Cuscuta* spp. WITH RESISTANT HOSTS

*Cuscuta* spp. have a broad host spectrum and therefore lots of possible plant “victims” to sustain them, but there are a few plants that successfully fend off *Cuscuta*. Many *Cuscuta* species are not able to infect monocotyledonous plants, probably for anatomical reasons such as the arrangement of vascular bundles or incompatibility of signals that are important for forming interspecies connections of vascular strands (Dawson et al., 1994). Nevertheless, this incompatibility seems quite passive and exceptions exist—*Cuscuta australis*, for instance, is able to infect monocotyledonous plants (**Figure 1B**).

So, are there plants that can specifically detect and “actively” defend themselves against parasitic dodder? Examples of plants that appear to mount an active defense against *Cuscuta* include Malvaceae *Gossypium hirsutum* and *Hibiscus rosa-sinensis*, neither of which can be penetrated by *Cuscuta* spp. In these plants, connection of parasitic haustoria or searching hyphae to the host vascular bundles is blocked by a kind of wound tissue, and the parasitic cells of the searching hyphae—and finally the parasite—die off (Capderon et al., 1985).

**FIGURE 2:**
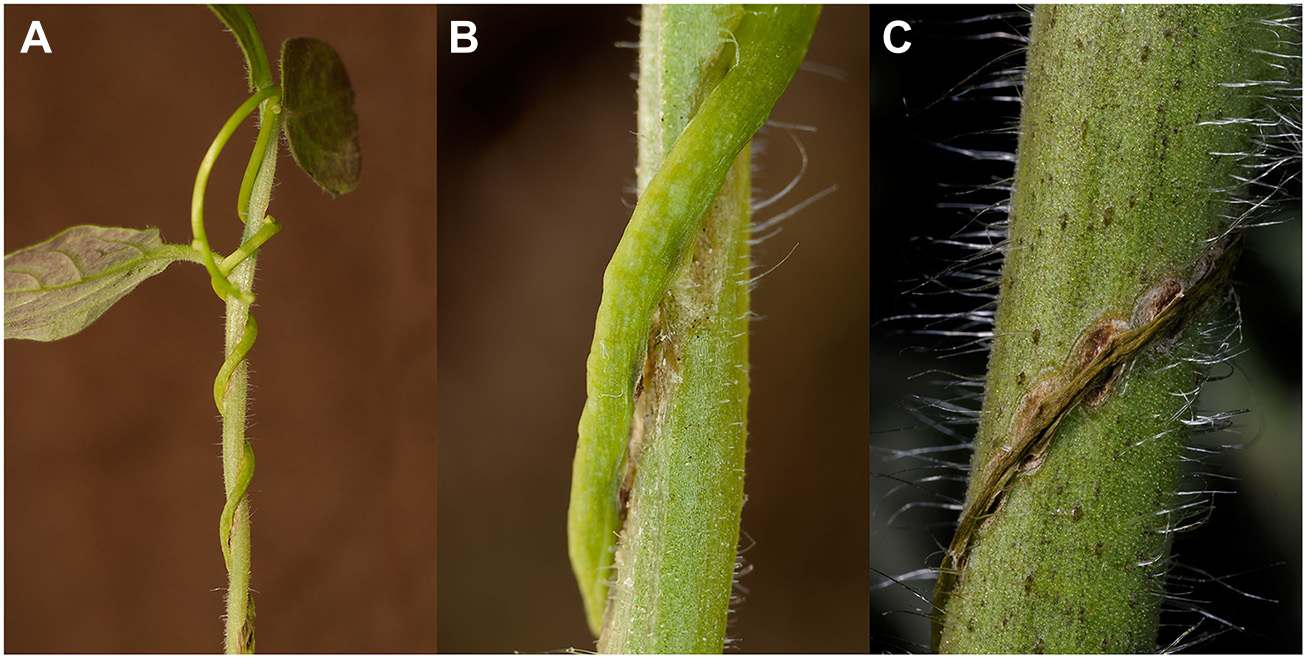
***Cuscuta reflexa* on its resistant host plant *Solanum lycopersicum* (cultivated tomato). (A)** *C. reflexa* ~7 days after initial contact to the tomato shoot. **(B)** Enlarged detail of **(A)** shows the secondarily modified tissue of tomato at the penetration sites next to the parasitic haustoria. **(C)** *C. reflexa* dies off ~14 days after initial contact with tomato; the secondarily modified tissue of tomato reminds of a kind of ‘hypersensitive response’ (HR).

**FIGURE 3:**
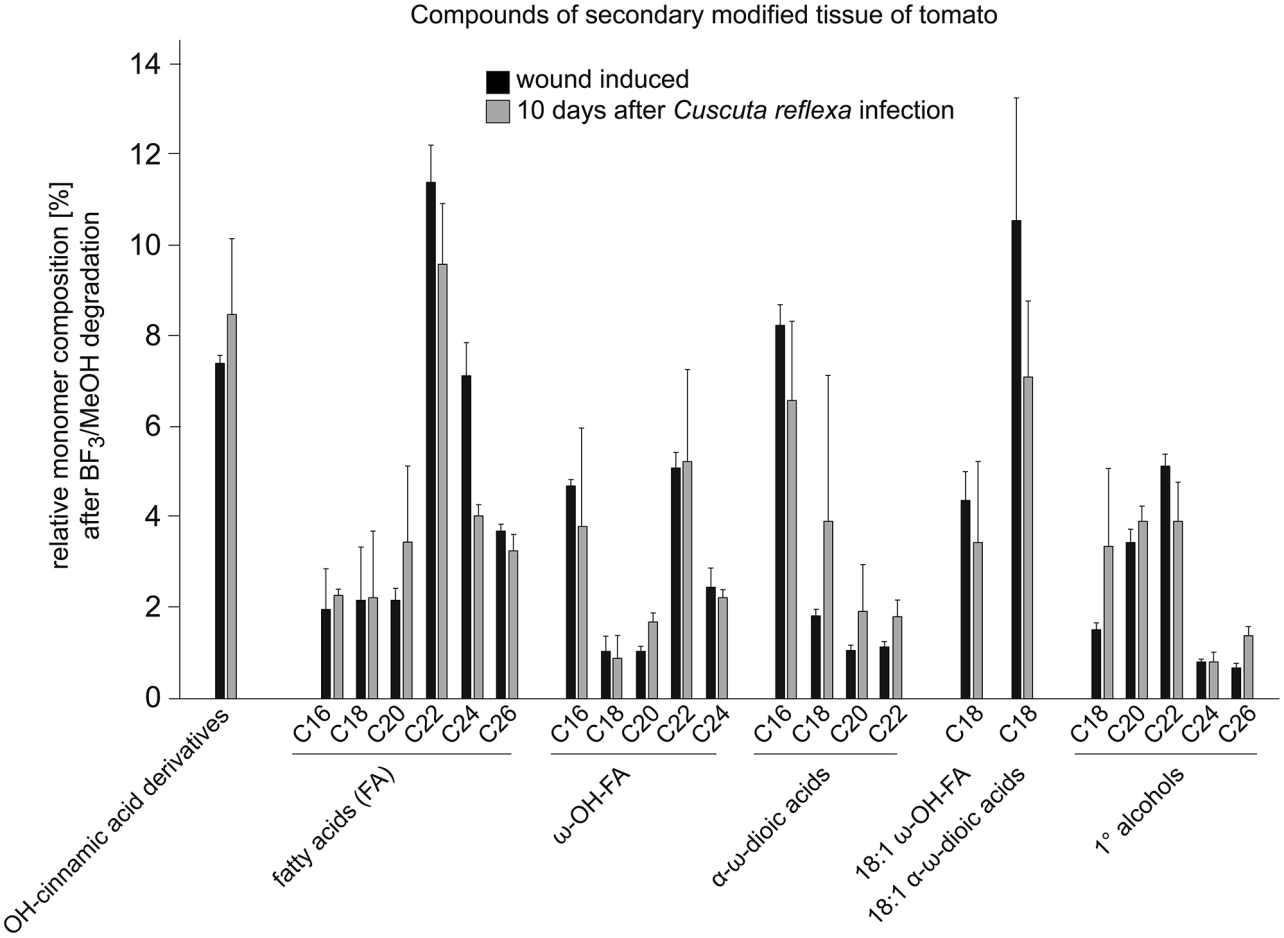
**Relative monomer composition of the secondarily modified tomato tissue.** The constituents were isolated from tomato shoot tissue infected with *C. reflexa* for ~10 days (for example see **Figure 2C**). Tissue was treated and degraded by BF_3_-methanolysis as described (Supplementary information, Leide et al., 2012) and analyzed via gas chromatography followed by mass spectrometry. Results (gray bars) were compared to those obtained after similar treatment of ‘wounding tissue’ (black bars; tomato stem ~10 days post wounding). Values are given in % of total constituents and represent means of measurements from three independent samples; error bars indicate SD.

Signs of an active resistance reaction can also be observed during the interaction of *C. reflexa* with the cultivated tomato (*S. lycopersicum*; Ihl et al., 1988; Albert et al., 2004, 2006; Runyon et al., 2010); at the end of the attachment phase, about 3–5 days after the parasitic prehaustoria have formed, epidermal host cells at the contact sites elongate strongly and burst (Ihl et al., 1988; Sahm et al., 1995; Löﬄer et al., 1999; Werner et al., 2001; Albert et al., 2004). This expansion of cells requires the expression of genes encoding proteins known to be involved in cell elongation, such as aquaporins (Werner et al., 2001) or proteins important for cell-wall modifications and restructuring like Xyloglucan endotransglycosylases/hydrolases (LeXTH; Albert et al., 2004). Cell elongation and gene expression are controlled by auxin, which strongly increases in the parasitic prehaustorium and in the epidermal tomato cells (Löﬄer et al., 1999). At the contact sites, tomato cells of the hypodermal tissue also elongate, and parenchymal cortex cells show increased cell division, resembling an extra meristem (Ihl et al., 1988). However, these responses are reminiscent of plant developmental processes rather than typical resistance responses. In particular, the involvement of an XTH, the expression of aquaporins and the growth-related phytohormone auxin indicate a developmental program that is probably also important for the infection of susceptible hosts. However, in parallel, *C. reflexa* appears to induce a defense program in the tomato, in which tomato cells at the infection site secrete soluble phenylpropanoids and show an increased accumulation and activity of peroxidases—enzymes that are important for linking phenylpropanoids with other components of the cell wall such as proteins, pectins, or cellulose fibers (Löﬄer et al., 1995, 1997; Sahm et al., 1995). This modified cell wall is thought to seal the infection site, preventing penetration of the tomato by *C. reflexa*. At the contact sites of *C. reflexa* haustoria with the tomato stem, a browning of tomato tissue, clearly visible by eye, appears within 5–7 days of interaction (**Figure 2**). Under UV light, this brownish tissue fluoresces strongly in a manner that indicates occurrence of phenylpropanoids in lignified plant tissues. When further analyzed for chemical composition by gas chromatography followed by mass spectrometry (GC-MS), this modified tissue, much like wound tissue, was indeed found to contain substances belonging to the phenylpropanoids (such as dihydroxy-cinnamic acid derivatives) as previously described (Löﬄer et al., 1995, 1997; Sahm et al., 1995), as well as long-chain (C18–C26) fatty acids (FA), ω-OH-FAs, α-ω-dioic acids and primary alcohols (**Figure 3**; Albert, 2005). Together with the phenylpropanoids, these hydrophobic long-chain components are known to become cross-linked within the cell wall, building the “wound suberin” of the secondarily modified plant cell wall (Bernards, 2002;Leide et al., 2012).

In summary, in direct contact with *C. reflexa* haustoria, epidermal tomato cells elongate and die following a hypersensitive-type response (Ihl et al., 1988) while a secondarily modified tissue is formed in the hypodermis to protect against haustoria penetration. Finally, the parasite dies off about 14 days after first contact (**Figure 2C**).

**FIGURE 4:**
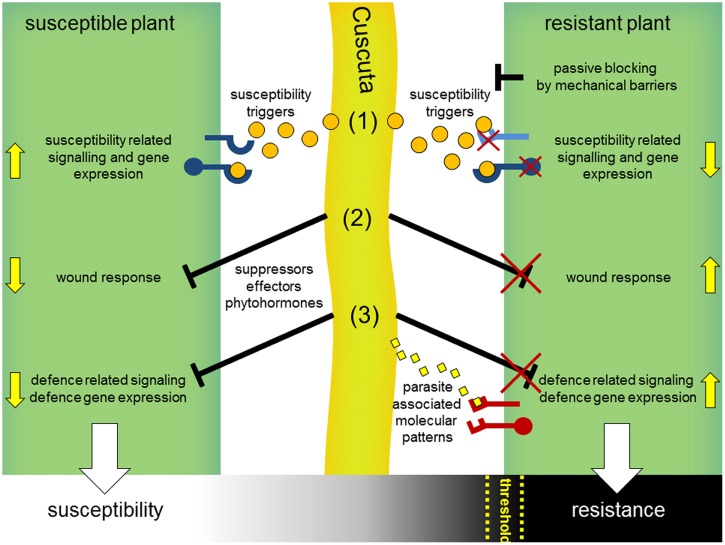
**Hypothetical model for interaction mechanisms of parasitic *Cuscuta* with susceptible and resistant host plants.** *Cuscuta* (**middle**, light green) attacking susceptible plants **(left)** release susceptibility triggers (orange dots) including common phytohormones and yet unknown signals, which get perceived by host plant receptors (blue) and consequently manipulate hosts to set on susceptibility-related responses and gene expression (1). In parallel, wound-related (2) and defense-related responses (3) that occur in the context of host penetration might get blocked by yet unknown suppressors. Resistant plants **(right)** might prevent a parasitic attack passively, e.g., by reinforced cell walls as mechanical barriers or by non-responsiveness to susceptibility triggers (1). Incompatibility could also result from a deficient blocking of the host wound (2) or defense response (3). In analogy to the perception of microbial pathogens, defense reactions might be actively triggered by host immunoreceptors (PRRs, red) that detect specific parasite-associated molecular patterns (3) (yellow diamonds) or secondary generated (e.g., by parasitic hydrolytic enzymes) damage-associated molecular patterns (DAMPs). Susceptibility or resistance of host plants might be a sum of these interaction processes and, rather than black and white, appears to be a gradual process occurring in many plants. Thus, a certain threshold for the strength of defense reactions might be passed by host plants for a successful resistance against dodder.

Is this resistance to *Cuscuta* due only to a wound response? This is called into doubt by the observation that these wound symptoms, and induction of the two stress hormones SA and JA, also occur in the tomato after interaction with *Cuscuta pentagona*, which can successfully infect this host. *C. pentagona*, however, induces visible HR symptoms in tomato as well and can grow better on transgenic tomato plants expressing a gene for a salicylate hydroxylase (*nahG*). In these *nahG* plants, the HR symptoms during the interaction with *C. pentagona* seem clearly reduced, indicating a correlation of SA level and HR symptoms during a *Cuscuta* attack (Runyon et al., 2010). Thus, a resistance mechanism including a host wound response or not, might be the sum of several factors and might depend on a threshold of the strength of diverse host defense responses that has to be passed for winning this battle (**Figure 4**). Indeed, the strong resistance reaction of cultivated tomato (*S. lycopersicum*) seems very specific to *C. reflexa*; several other *Cuscuta* species can successfully infect tomato, including *C. pentagona, C. suaveolens*, and *C. europaea* (Jiang et al., 2013b; Ranjan et al., 2014). In addition, wound responses at infection sites are not restricted to *S. lycopersicum* but also occur in nearly all host plants, so that wounding may not be sufficient to account for the complete resistance observed in the *C. reflexa–S. lycopersicum* interaction. Also possible, dodder might actively block wound and defense responses when infecting susceptible plants by suppressors and an unsuccessful suppression of these responses could lead to defense or resistance as well (**Figure 4**).

The resistance reactions of tomato to *C. reflexa* attack may go beyond wound responses to include other reactions that are commonly observed after infection by microbial pathogens (**Figure 4**). Plant hosts can detect microbial pathogens in different ways, either directly by perception of MAMPs or indirectly by perception of damage-associated molecular patterns (DAMPs) that arise during attack by pathogens. DAMPs comprise a range of different molecules, from solubilized fragments of cell-walls or plasma membrane constituents to cytoplasmic proteins and ATP (Küfner et al., 2009; Ottmann et al., 2009; Bartels et al., 2013; Choi et al., 2014). DAMPs and MAMPs both signal “danger” to the host plant, and both types of signal are generally perceived via receptor proteins at the surface of the plant cells (Dodds and Rathjen, 2010; Böhm et al., 2014; Macho and Zipfel, 2014). In addition, plants have further systems for pathogen detection that function mainly intracellularly and depend on an array of resistance proteins (Lanfermeijer et al., 2003). Typically, these resistance proteins trigger strong HR-symptoms which look cortically similar to those induced by *C. reflexa* on a tomato stem and thus could provide a comparable mechanism for the observed resistance. Together with membrane bound receptors (e.g., LRR–RLPs), the intracellular NBS–LRR proteins are known to recognize pathogen-derived avirulence (avr) proteins. Famous examples therefore identified in tomato might be presented by Cf-receptor proteins and their recognized AVR-protein ligands, encoded by the avr-genes of the fungus *Cladosporium fulvum* (Cf; De Wit et al., 2009; Wulff et al., 2009). The perception of AVR4 by its cognate receptor Cf-4 follows the concept of a gene-for-gene interaction and already a single point mutation in the avr-gene leads to a mismatch and consequently to a lack of resistance (Joosten et al., 1994). This gene-for-gene model has already been observed and defined earlier (Flor, 1956, 1971) and has been used in breeding efforts to select pathogen-resistant genotypes of single plant species for a very long time.

About 10 years ago, this strategy was also applied to screen different commercial hybrid tomato varieties for their resistance responses to lespedeza dodder. In this study, three varieties have been identified which exhibited tolerance (resistance) to the parasite compared to the fully susceptible variety “Halley 3155.” The resistance against this dodder species seems to be incomplete since the dodder haustoria still could penetrate the tomato stem in many cases. However, in field studies, 75% less lespedeza dodder attachments were observed on tolerant varieties, and dodder growth was reduced by more than 70% (Goldwasser, 2001). The highly variable resistance of tomatoes which seems to appear at various strengths argues for a phenomenon driven by quantitative resistant traits. Supposably, resistance genes, or their encoded proteins, respectively, act synergistically to display a full resistance. As soon as one component lacks, resistance symptoms might be visible on tomato stems without committing full resistance.

Within other plant genera genotypes resistant against *Cuscuta* spp. were identified as well. After a large-scale greenhouse experiment, a set of chickpea genotypes (*Cicer arietinum*) was screened for the resistance response to *Cuscuta campestris* (Goldwasser et al., 2012). While most of the chickpea varieties displayed susceptibility, two genotypes were identified which clearly showed resistance against *C. campestris* and 80% of these chickpea plants were fully resistant. This resistance mechanism, however, seems to be different from the one observed for tomato plants since it lacks a HR-like response or lignification close to the haustoria attachment sites. The phenomenon was described as a kind of passive repellent of the parasite haustorium prior to penetration and consequently starvation of *C. campestris* (Goldwasser et al., 2012). Similar resistance mechanisms of haustoria penetration rejection have been reported for crops against root parasites of the *Orobanche* spp. (Goldwasser et al., 2000; Perez-de-Luque et al., 2006, 2008; Yoder and Scholes, 2010). For example, in chickpea lines resistant against *Orobanche crenata* Forsk., the ingrowth of haustoria was blocked before reaching the central cylinder of the host roots (Rubiales et al., 2003). The resistance response of cowpea cultivar B301 to race 3 (SG3) of *Striga gesnerioides* is characterized by an inability of the parasite to penetrate the endodermis and by necrosis of the host tissue at the point of attachment as well. In 2009, a gene conferring this resistance against *Striga* was identified in cowpea which encodes for an intracellular resistance protein of the CC-NBS-LRR family (Li and Timko, 2009). Since the resistance of diverse cowpea cultivars against different *Striga* species is race specific, the resistance mechanism clearly follows a gene-for-gene interaction model (Li and Timko, 2009; Li et al., 2009). Although root-parasites of the Orobanchaceae infect an anatomically different organ than shoot parasites of the genera *Cuscuta*, the cellular resistance mechanisms might show parallels. A few inspiring works were reviewed by Yoder and Scholes (2010).

At present it is not clear whether resistance of tomato to *C. reflexa* depends on one or more specific resistance proteins. It is also unclear as yet whether *C. reflexa* produces DAMPs or whether it releases molecular patterns that are perceived by tomato in a manner analogous to MAMPs or avr proteins. However, such mechanisms are imaginable and the question of how *C. reflexa* is perceived by the tomato immune system is one of the topics currently under investigation in our lab.

## CONTROLLING *Cuscuta* spp. INFECTIONS

*Cuscuta* spp. has a wide host range, including many cultivated crops such as tomato, tobacco, clover, and dicotyledonous weeds as well as trees and shrubs, but only a few grasses or monocotyledonous weeds (Dawson et al., 1994; Albert et al., 2008). Hosts are attacked non-specifically and sometimes even simultaneously, and one crop species may serve as a host for several dodder species (Cudney and Lanini, 2000). Depending on the infected plant species, *Cuscuta* infestation has more or less severe effects on the growth and reproduction of its host. Rather than causing host death, *Cuscuta* infestation seems to weaken host plants and to render them more susceptible to secondary diseases such as infection by microbes or insect and nematode infestation (Lanini and Kogan, 2005).

Dodder seeds are easily spread by animals and man, notably through international exchange of contaminated host seeds or movement of equipment or soil (Cudney and Lanini, 2000). Once established in a field, it can be problematic to get rid of *Cuscuta*. Preventive measures such as crop rotation with non-host plants, delaying planting until fall, use of resistant varieties and use of herbicides are effective only to an extent (Parker, 1991); once *Cuscuta* is attached to its host, containment becomes difficult (Lanini and Kogan, 2005). The use of genetically modified, herbicide-resistant crops has been tested but has proved unpromising (Nadler-Hassar and Rubin, 2003). The protein PAT (phosphinothricin acetyl transferase), which confers resistance to the herbicide glufosinate, has been shown to traffic between herbicide tolerant soybeans and *Cuscuta pentagona* and is therefore not an option for controlling dodder (Jiang et al., 2013a). As none of the described strategies is 100% effective, and given the invasion by new *Cuscuta* species—e.g., *C. reflexa* (giant dodder), as introduced in California and New Zealand (Rejmanek and Pitcairn, 2002)—further research is required.

Promising future strategies for the control of parasitic plant infestations include transgenic plants expressing specifically designed small RNAs (Roney et al., 2007; Runo et al., 2011, 2012; Alakonya et al., 2012). This biotechnological approach of cross-species transfer of small RNAs has been shown to work well for control of *Cuscuta* infestations. Transgenic host plants expressing phloem-specific RNAi-constructs for SHOOT MERISTEMLESS-like (STM), a protein that plays an essential role in parasitic haustoria development, were infected with *Cuscuta pentagona*. The small RNAs successfully moved from the host into the *C. pentagona* haustoria, where they interfered with haustoria growth and consequently inhibited haustorium development, so reducing the infection (Alakonya et al., 2012). Further useful and promising strategies might include the control of parasitic enzymes essential for the *Cuscuta* infection process, such as blocking the infestation-specific cysteine protease Cuscutain by external application of its intrinsic inhibitor peptide in high excess (Bleischwitz et al., 2010). While promising, these approaches require improvement and optimization to reach the standards of agronomical application. Additionally, screening for resistant genotypes of certain crop species as described (Goldwasser, 2001; Goldwasser et al., 2012), breeding of resistant varieties and the transfer of resistance genes from one species (e.g., tomato) to crop plants may offer further means of controlling *Cuscuta* infection.

## CONCLUSION AND PERSPECTIVES

To establish strategies to control parasite growth and restrict the spread of *Cuscuta* spp. in crop fields, it is important to learn more about this pest, studying its life cycle, its development, and its molecular mechanisms of infection. Using a next-generation RNA sequencing platform, two very recent articles have focused on expressed genes of *Cuscuta* during the infection process as well as in growth and development (Jiang et al., 2013b; Ranjan et al., 2014). Jiang et al. (2013b) focused on two *Cuscuta* species, *C. pentagona* and *C. suaveolens*, and were able to provide more than 46,000 isotigs and contigs for each species. Interestingly, after comparing the datasets with sequence-libraries of other parasitic plants such as *Triphysaria*, *Orobanche,* and *Striga*, they could identify a set of ESTs that seem to be shared exclusively by parasitic plants. Ranjan et al. (2014) isolated RNAs of *Cuscuta pentagona* at different stages (i.e., from seeds, seedlings, vegetative strands, prehaustoria, haustoria, and flowers) and used them for *de novo* assembly and transcriptome annotation. These transcript pools provided insights into the transcriptional dynamics during dodder development as well as parasitism, and helped to identify gene categories involved in the infection processes of diverse stages (Ranjan et al., 2014). The use of these modern sequencing methods offers an overview and could nicely correlate the expression of certain genes during certain phases and processes in the life cycle of *Cuscuta*. However, these pioneering studies must be broadened through detailed analyses of molecular mechanisms, and further comprehensive studies of individual genes and their proteins will be necessary in order to understand the molecular mechanisms of infection processes. In particular, the identification of genes or proteins with key functions in mediating susceptibility or resistance to *Cuscuta* infestation will be of great importance. For example, identification of the receptors that perceive parasitic signals and initiate a “susceptibility” program in the host plants, as well as the signals secreted by the *Cuscuta* haustorium during the infection process, will be essential to understanding the basis of the *Cuscuta*-host interaction and may thus advance knowledge about plant–plant dialogs in general. From this foundation, new perspectives for controlling parasitic pests may evolve.

## Conflict of Interest Statement

The authors declare that the research was conducted in the absence of any commercial or financial relationships that could be construed as a potential conflict of interest.

## SUPPLEMENTARY MATERIAL

The Supplementary Material for this article can be found online at:
