# Supplementary material for: Parasitic plants of the genus Cuscuta and their interaction with susceptible and resistant host plants
Source: Front Plant Sci. 2015 Feb 4;6:45. doi: 10.3389/fpls.2015.00045 (PMC4316696; doi:10.3389/fpls.2015.00045)
Supplement: Supplementary file 1 [file Data_Sheet_1.DOCX]

**Supplementary information**

**Biochemical analysis of the secondarily modified tomato tissue (**[**Leide et al., 2012**](#_ENREF_50)**) (modified from (**[**Albert, 2005**](#_ENREF_2)**)**

**Sample preparation.** About 7–10 days after the first contact between *C. reflexa* and *S. lycopersicum*, the parasite was mechanically removed and the brownish tomato tissue (see also Fig. 2) was cut out from the tomato shoot with a scalpel. Remaining unmodified tissue was enzymatically removed (0.1% cellulase, 0.1% pectinase, 1 mM sodium azide) by shaking (10 rpm) at room temperature for ~3 days; eventually associated xylem fibres were removed with forceps. The obtained tissue samples were dried on a filter and were either stored in a sample glass tube or directly used for the BF_3_ methanolysis.

For wound tissue, tomato stems were treated with a wire brush and samples were collected 7 days post-wounding; samples were treated the same way as described above.

**BF_3_ methanolysis.** Samples were washed 2x with Chloroform and dried. 1–1.5 mg of material were taken out and 1.5 ml of a 10% BF_3_/MeOH solution (Fluka; boron trifluoride, ~ 1.3 M in methanol) was added. Tubes were closed (screw caps with cartridge seal), incubated overnight at 70 °C and cooled to room temperature the next day. The reaction solution was transferred to a new tube, the remaining sample was washed 2x with 1 ml Chloroform and the washing solution was unified with the reaction solution. After addition of 20 µg Dotriacontan (=C32) as internal standard, samples were washed 2x with saturated NaCl solution; the upper aqueous phase was discarded with a Pasteur pipette. The organic phase was dried with Na_2_SO_4_, filtered and concentrated to a volume >100 µl. 100 µg (=100 µl) of sample were transferred to a reaction tube and again concentrated for 1–2 h at 60 °C with gaseous Nitrogen to a very small volume (almost completely dry). Samples were supplied with 10 µl BSTFA and 10 µl Pyridine, derivatized for 60 minutes at 70 °C and cooled to room temperature for 20 min. All samples were solved in 100 µl Chloroform and 50 µl were transferred to analysis-tubes for gas chromatography (FID) followed by mass spectrometry (MS).

**Gas chromatography program**

starting temp: 50 °C

starting time: 4 min

temperature program rate (°C/min) final temp (°C) final time (min)

level 1 10 150 2

level 2 3 320 30

level 3 no no no

starting pressure: 50 kPa

starting time: 70 min

pressure program rate (kPa/min) final pressure (kPa) final time (min)

level 1 10 150 30
